# Comparative analyses of holder pasteurization vs. HTST pasteurization for donor milk: a cost-minimization study applicable to human milk banks

**DOI:** 10.1186/s13006-023-00557-1

**Published:** 2023-04-14

**Authors:** Diana Escuder Vieco, Jorge Arenas Vidal, Paula Rojas García, Marino J Gónzález, Nadia Raquel García Lara, Carmen Rosa Pallás Alonso

**Affiliations:** 1 Servicio de Neonatología, Banco Regional de Leche Materna, Hospital Universitario 12 de Octubre, Instituto de Investigación i+12, Madrid, Spain; 2 BioSerentia Group, Madrid, Spain; 3 Department of Economics, University of La Rioja, Logroño, Spain; 4 Center for Biomedical Research of La Rioja (CIBIR), Logroño, Spain; 5 Servicio de Neonatología, Instituto de Investigación i+12, Banco Regional de Leche Materna, Hospital Universitario 12 de Octubre, Universidad Complutense de Madrid, Madrid, Spain

**Keywords:** Donor milk, Cost-minimization analysis, Pasteurization, Human milk banks, Opportunity cost

## Abstract

**Background:**

High-temperature short-time (HTST) pasteurization (72–75 °C, 15 s) is an alternative treatment to traditional Holder pasteurization (HoP) (62ºC, 30 min) for donor milk. HTST pasteurization guarantees the milk’s microbiological safety and retains more of its biologically and nutritionally active compounds, but the cost of implementing this technology for a human milk bank is unknown.

**Methods:**

A cost-minimization study was carried out on the facilities of a regional human milk bank in a public hospital. Total production costs (fixed plus variables) were quantified using HTST pasteurization and HoP in three hypothetical scenarios: (1) costs of the first 10 L of pasteurized milk in a newly opened milk bank; (2) costs of the first 10 L of pasteurized milk in an active milk bank; and (3) costs using the maximum production capacity of both technologies in the first two years of operation. The following costs were analyzed: health care professionals, equipment and software, external services, and consumables.

**Results:**

In scenario 1, the total production costs were € 228,097.00 for the HTST method versus € 154,064.00 for the HoP method. In scenario 2, these costs were similar (€ 6,594.00 for HTST pasteurization versus € 5,912.00 for HoP). The cost of healthcare professionals was reduced by more than half when pasteurization was carried out by the HTST method versus the Holder method (€ 84.00 and € 191.00, respectively). In scenario 3, the unit cost of milk pasteurized by the HTST method decreased from the first to the second year by 43.5%, while for the HoP method, it decreased by 30%.

**Conclusions:**

HTST pasteurization requires a high initial investment in equipment; however, it provides a significant minimization of production costs in the long term, pasteurizes large quantities of donor milk per working day and achieves a more efficient management of the time of the health care professionals in charge of the bank’s operation compared to HoP.

## Background

Breastfeeding is the optimal type of feeding for newborns during the first six months of life [1]. There are numerous studies on the benefits attributed to breastfeeding for the health of the mother and the development of the newborn in both the short and long term [2, 3]. In addition, the promotion of breastfeeding is one of the strategies that minimizes economic losses for society [4] with an estimated potential savings for the Spanish National Health System of more than 5.6 million euros for each percentage point increase in exclusive breastfeeding rates in Spain during 2014 [5].

However, there are situations in which the mother’s own milk (MOM) is not available or is in short supply to meet the nutritional requirements of the newborn. On these occasions, donor human milk (DHM) processed in human milk banks (HMBs) is the best alternative, especially for premature or sick newborns [6]. The use of DHM is associated with a reduction in the incidence of necrotizing enterocolitis (NEC), protection against late-onset sepsis and improved feeding tolerance compared to formula milk in this high-risk group of infants [7] which in cost-effectiveness studies has resulted in significant economic savings. Johnson et al. found that MOM + DHM was associated with US$15,555.00 lower costs per infant (*p* = .045). Additionally, NEC was associated with a US$66,015.00 higher cost per infant [8]. In addition, opening HMBs in neonatal units has been shown to increase breastfeeding rates [9].

To ensure microbiological safety, DHM is pasteurized in most HMBs to kill all non-spore forming and potentially pathogenic microorganisms. At present, low-temperature long-time (heating at 62.5 °C for 30 min) pasteurization, also known as “Holder” pasteurization (HoP), is the heat treatment most commonly applied to DHM [10]. This treatment can be carried out in thermal baths with constant agitation or in validated semiautomatic pasteurizers designed for this purpose [11].

Unfortunately, this heat treatment has been shown to have a negative impact on some of the biologically active or nutritional compounds present in DHM [12]. For this reason, the European Association of Human Milk Banks (EMBA) recommends the research and establishment of alternative treatments to HoP [10]. Various laboratory prototypes have been described for the treatment of DHM using high-temperature short-time (HTST) pasteurization, high-pressure processing or ultraviolet irradiation [13]. Recently, a system for continuous pasteurization using the HTST method (72ºC, 10– 15 s) adapted to the real conditions of a regional milk bank has been described [14]. This system is better at preserving the functionality of some nutritional and biologically active components of DHM compared to HoP [15, 16]. It has also been reported that high-pressure processing is a potentially more beneficial method of DHM preservation than the HoP method. However, in some settings it has been found to be approximately seven times more expensive [17].

The administration of DHM is a low-cost intervention compared to many others for the care of hospitalized infants [18]. For this reason, it is desirable to develop and implement alternative solutions to the traditional pasteurization method that improve milk quality but at the lowest possible cost. To date, there are no studies on the cost of implementing the processing of DHM with an alternative treatment such as HTST pasteurization in HMBs. Therefore, the objective of this study was to perform a cost analysis to quantify the alternative of using HTST pasteurization versus the standard HoP.

## Methods

### Study selection

A cost minimization analysis was performed to estimate the cost of processing the first 10 L of DHM by HoP and HTST pasteurization. This initial quantity was selected because it is the maximum volume that can be pasteurized by HoP using thermal baths in the facilities of our HMB (Regional Human Milk Bank, Community of Madrid, Spain) daily. Moreover, this quantity matches the flow rate validated (10 L / h) for the continuous HTST pasteurizer.

Both alternatives have been shown to be valid options in the effective pasteurization of DHM; however, it is unknown whether the clinical effects of administering DHM by one method or the other are better in preterm infants. Therefore, it is assumed that the health effects do not differ significantly between the alternatives compared, so that the option that represents the least cost has the best “value for money” [19].

### Data extraction

**Fig. 1:**
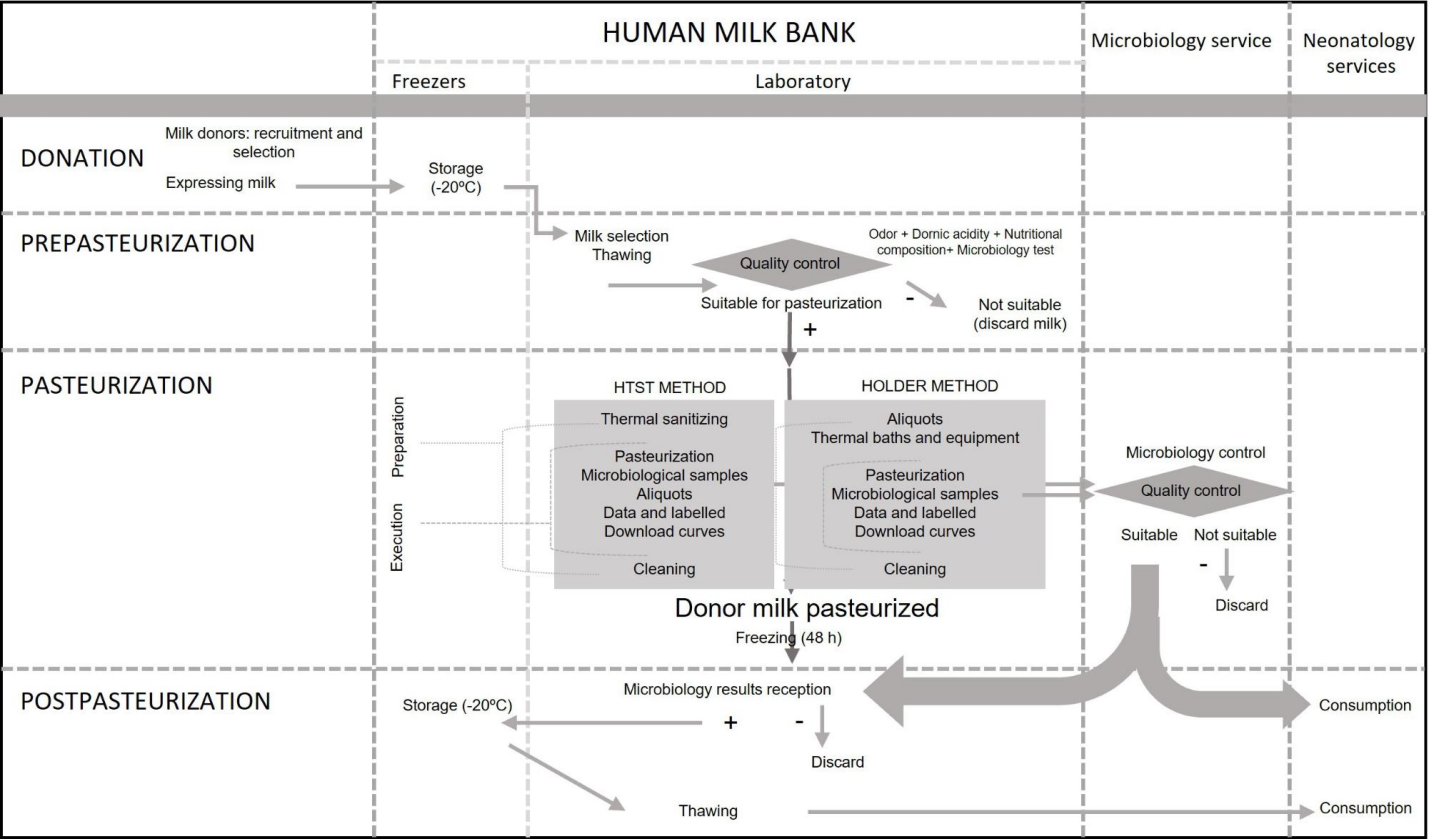
Functional map of the human milk bank’s processes and subprocesses

To extract data to quantify costs, a functional map of processes and subprocesses was constructed. It included three stages: pre-pasteurization, pasteurization and post-pasteurization (Fig. 1).

In the pre-pasteurization stage, DHM to be pasteurized was selected and subjected to rigorous microbiological, biochemical and nutritional quality controls. In the post-pasteurization stage, the milk was stored frozen at -20ºC until its distribution. In the pasteurization stage, HoP or HTST pasteurization was applied, each involving different costs. The analysis also included the costs of the pre-pasteurization and post-pasteurization stages to quantify the total costs of opening an HMB. The costs of recruitment and selection of donors (blood tests or nurse’s time for the interview with the donor) as well as expression (containers, breast pumps or other consumables) and transport of DHM from home to the hospital were not considered. In addition, other indirect costs were not included in this study because they are common to both pasteurization processes.

### Costs

The public payer perspective was adopted and included all direct health care costs generated in a public hospital from the pre-pasteurization stage until the end of the post-pasteurization stage. These costs included those generated by health care professionals, mainly linked to their salary. The professional categories involved in the stages studied had the following salaries for professional practice in the Madrid Health Service: laboratory technician (€ 24,468.00 gross per year) or medical supervisor (€ 45,944.00 gross per year). The health care professional cost for each subprocess was calculated on the basis of the time dedicated to it in minutes and multiplied by the cost per minute of its economic income. The times were measured with stopwatches during routine activity in the HMB. These costs were considered as an opportunity cost as time and talent of professionals could be dedicated to other tasks.

Costs generated by the use of infrastructure, equipment and material resources were also included. The equipment category included the costs corresponding to those apparatus, equipment or devices of any nature that were inventoriable and were subject to accounting depreciation. This category also included software licenses and computer databases. The prices of the equipment were those provided directly by the manufacturers and / or distributors according to the selling price in Spain (PVL) and served at the hospital.

The material category included all material not subject to accounting depreciation, inventoriable or not, typically glass or plastic, sterile or nonsterile, most of which is consumed with the pasteurization of batches of DHM daily. The prices of consumables and other material were obtained from information provided by the Purchasing Department, the Management Department and the Medical Department of the hospital.

### Scenarios

To illustrate the economic comparison between the use of HTST pasteurization and HoP, three hypothetical scenarios were proposed.

Scenario 1 quantified the costs of pasteurizing the first 10 L of DHM by HTST pasteurization and HoP for a newly opened HMB. All the categories included in this scenario were considered: health care professionals, acquisition of equipment and software, external services, materials and consumables.

Scenario 2 quantified the costs of pasteurizing the first 10 L of DHM using both techniques when there was already an active HMB that had made the investment in equipment and software. In this case, only the costs of health care professionals, external services and materials and consumables were incurred for each pasteurization method.

In scenario 3, costs were compared and quantified taking into account the maximum quantities of pasteurized milk that could be obtained using both technologies during the first two years of operation of our HMB. The maximum daily quantity that could be processed by the HTST method was established as 20 L of milk, from which 18 L of pasteurized milk could be obtained. This difference in volume corresponded to the dead volume inherent to the design of the HTST pasteurizer. Taking this into account, a total production of 90 L per week was estimated, equating to 360 L per month. Assuming 11 net months of continuous pasteurized milk production, 3,960 L could be obtained the first year, and a total of 7,920 L could be obtained the second year. As the maximum daily quantity that can be pasteurized by HoP, where thermal baths are used for heating and cooling, is 10 L of milk, we would obtain a total of 50 L per week, 200 L per month and, assuming 11 net months of continuous production of pasteurized milk, 2,200 L per year and 4,400 L in two years.

For both pasteurization methods, the investment required to obtain the maximum amount of pasteurized milk in the first year of operation of the HMB (3,960 L per year by the HTST method and 2,200 L per year by the HoP method) should be centered on acquiring the equipment and software, the inventoriable material and hiring the necessary health care professionals. In the second year, the investment in equipment, software and inventoriable material has already been made, and only the investment in health care professionals and annual maintenance services should be provided.

### Data analysis

The extracted data were entered into Excel 2020 (Microsoft Corp, Washington, USA) and analyzed.

## Results

### Scenario 1

The total cost (fixed plus variable) of processing the first 10 L of DHM for a newly opened HMB by the HTST method was € 228,097.00, and it was € 154,064.00 by the Holder method using thermal baths, with the latter method saving € 74,033.00 (Table 1).

**Table 1:** Total costs of processing the first 10 L of DHM using the HTST method or the Holder method in a newly opened human milk bank

		Processes					Total processes	
**Costs (€)**	Pre-pasteurization	HTST pasteurization	HoP	Post-pasteurization			HTST method	Holder method
Health care professionals	94,880.00	94,880.00	94,880.00		94,880.00		94,880.00	94,880.00
Equipment and software	39,042.00	85,750.00	12,806.00		1615.00		126,407.00	53,463.00
External services	1050.00	3010.00	1220.00		60.00		4120.00	2330.00
Materials and consumables	1856.00	832.00	1533.00		2.00		2690.00	3391.00
Total	41,948.00	184,472.00	15,559.00		1677.00		228,097.00	154,064.00

Abbreviations: *DHM* Donor human milk, *HoP* Holder pasteurization, *HTST* High-Temperature Short-Time

The equipment and software category had the highest cost if pasteurization was carried out by the HTST method (€ 126,407.00) versus the HoP method (€ 53,463.00). On the other hand, the materials and consumables category had the highest cost for HoP (€ 3,391.00) versus HTST pasteurization (€ 2,690.00) (Table 1).

### Scenario 2

The total cost of processing the first 10 L of DHM in an active HMB using the HTST method was € 6,594.00 versus € 5,912.00 using the Holder method, the latter method saving € 682.00 (Table 2).

**Table 2:** **Total costs of processing the first 10 L of DHM using HTST method or Holder method in an active human milk bank**

Costs (€)	Pre-pasteurization	HTST pasteurization	HoP	Post-pasteurization
Health care professionals	61.00	84.00	191.00	0
Equipment and software	0	0	0	0
External services	1050.00	3010.00	1220.00	60.00
Materials and consumables	1856.00	832.00	1533.00	2.00
Total	2966.00	3926.00	2944.00	62.00

Abbreviations: *DHM* Donor human milk, *HoP* Holder pasteurization, *HTST* High-Temperature Short-Time

In this scenario, the external services category was the only one that had a higher cost for HTST pasteurization versus HoP (€ 3,010.00 and € 1,220.00, respectively).

In contrast, HoP carried out in thermal baths had a higher cost for the materials and consumables category and, especially, for health care professionals. In this sense, the opportunity cost for health care professionals was reduced by more than half when pasteurization was carried out using HTST pasteurization versus HoP (€ 84.00 and € 191.00, respectively). It was estimated that the medical supervisor profile spent 8.52% of time when HTST pasteurization was performed versus 10.44% when pasteurization was performed using HoP. For the laboratory technician profile, the time spent using HTST pasteurization was 91.48% versus 89.73% using HoP.

Regarding the times measured for each subprocess, in the case of HTST pasteurization, the complete cleaning of the equipment was the subprocess that consumed the most time (150 min), and the unloading and validation of the pasteurization curve consumed the least time (10 min). For HoP, the preparation of the aliquots before heat treatment was the most time-consuming process (165 min), while the preparation of the thermal baths (adjusting temperature and water level) for heating and cooling was the least time-consuming (10 min).

### Scenario 3

Assuming the maximum milk production that can be pasteurized using the HTST method and the HoP method, the average unit cost over a two-year period was calculated to be 58.53 € / L (€ 41.20 as fixed cost + 17.33 € / L as variable cost) for milk pasteurized using HTST pasteurization and 72,97 € / L (€ 57.07 as fixed cost + 15.90 € / L as variable cost) for milk pasteurized using HoP (Table 3).

**Table 3:** **Total costs of processing the maximum amounts of DHM by the HTST method or the Holder method during the first two years in our human milk bank**

Method	HTST method			Holder method		
Costs (€)	Year 1	Year 2	Year 1 + 2	Year 1	Year 2	Year 1 + 2
Health care professionals	94,880.00	94,880.00	189,760.00	94,880.00	94,880.00	189,760.00
Equipment and software	126,407.00	0	126,407.00	53,462.00	0	53,462.00
Maintenance services	4120.00	4120.00	8240.00	2330.00	2330.00	4660.00
Inventoriable material	2689.00	0	2689.00	3232.00	0	3232.00
Total fixed costs (€)	228,096.00	99,000.00	327,096.00	153,904.00	97,210.00	251,114.00
Maximum amount pasteurized (L)	3960.00	3960.00	7920.00	2200.00	2200.00	4400.00
Total fixed costs per liter (€ / L)	57.60	25.00	41.20	69.96	44.20	57.07
Total variable costs per liter (€ / L)	17.33	17.33	17.33	15.90	15.90	15.90
Unit cost (€ / L)	74.93	42.33	58.53	85.86	60.10	72.97

Abbreviations: *DHM* Donor human milk, *HTST* High-Temperature Short-Time

**Fig. 2:**
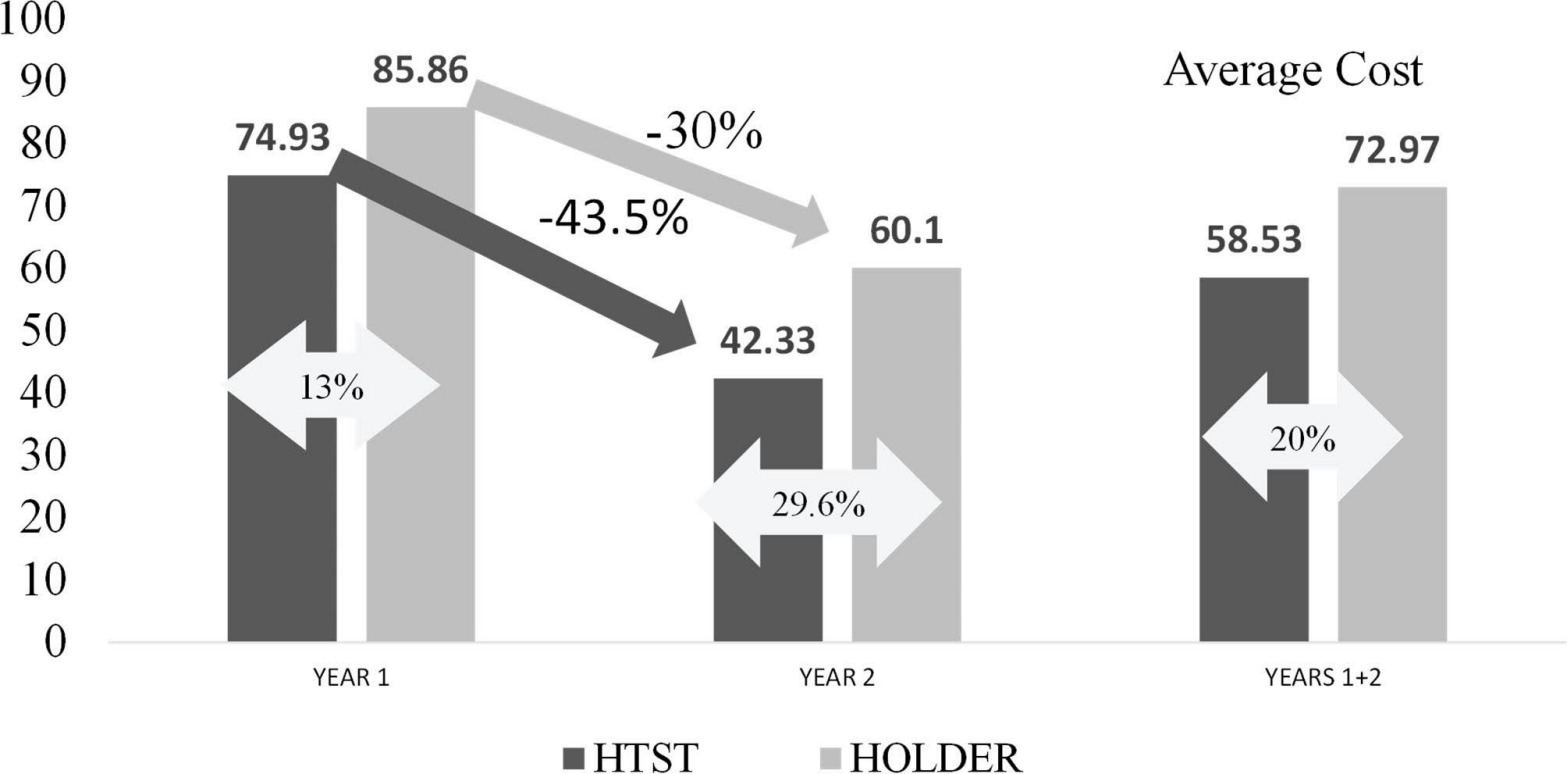
Unit costs (€ / L, %) of processing the maximum amounts of DHM by HTST pasteurization and HoP during the first two years in our HMB

The total unit cost in year 1 and year 2 for each method, as well as the percentages of cost minimization between these years, are shown in Fig. 2. Thus, the unit cost decreased from the first to the second year from 74.93 € / L to 42.33 € / L (43.5%) using HTST pasteurization, while the equivalent cost decreased from 85.86 € / L to 60.1 € / L (30%) using HoP.

## Discussion

In this study, a cost-minimization analysis was carried out to quantify the cost of processing DHM using the traditional HoP method and an alternative method such as HTST pasteurization. Overall, the cost of opening a regional HMB using HoP is less than opening one using HTST technology. However, once the investment in equipment and software is made, the costs are equivalent for both technologies, and health care professionals’ time is used more efficiently by the HTST method. In addition, if such pasteurization systems operate at their maximum production capacity, the unit cost of pasteurized milk is further minimized by HTST pasteurization compared to HoP.

The opening of HMBs is a strategic decision that has benefits for both the health of the most vulnerable newborns and for the promotion of breastfeeding, as well as a reduction in medical costs [20]. However, this decision requires a substantial economic investment, so it is interesting to know the long-term costs associated with the chosen pasteurization method.

An increasing number of human milk banks are being established around the world to facilitate the collection, processing and distribution of DHM. According to data from the European Milk Bank Association, there are 280 active milk banks [21], and HoP is the heat treatment of choice in most of them [11].

In this study, the cost of production of the first 10 L of milk for a newly opened HMB implementing HoP was € 154,064.00, and the average unit cost was 72.97 € / L during the first two years of operation at maximum production. The operation costs of HMBs across different countries are similar to or even higher than those published in this study. In China, Daili et al. found an annual production cost of US$156,923.00 and a unit cost of 168 US$ / L [22]. In Germany the total cost per year was € 92,085.02 for 300 L of DHM: 27% of this was material costs, 51% was personnel costs and 22% was other overheads; moreover, the average cost per liter was € 306.95, and staff time was 492 min per liter [23]. In Italy, human milk costs approximately € 130 / L [24]. In Taiwan, the processing fee of each liter of donor milk is approximately 170 US$ / L [25].

Implementing HTST pasteurization in a newly opened HMB involved a higher cost than implementing HoP (€ 228,097.00 versus € 154,064.00). This difference was mainly attributed to the equipment and software category, since the purchase of an automated pasteurizer for HTST treatment involves a higher initial investment in technology than the purchase of a standard thermal bath (devices that we use in our milk bank), which is the most economical option. Even so, in the event of acquiring semi-automatic pasteurizers in the future, it would be interesting to incorporate their results into the present comparative study by calculating the specific costs associated with them in the clinical routine equivalent to the other two procedures already studied. There would obviously be more initial investment, but it is expected that this would be compensated in part by savings in personnel time and higher annual productivity. Only then would it be possible to establish to what extent and in which cases the option of semi-automatic pasteurizers to cover punctuality or structural demands, of small or large quantities of milk, would be the choice as opposed to the HoP with thermal baths / HTST options.

In the case of an active HMB (scenario 2), the structural fixed costs were practically the same using both pasteurization methods. Variable costs, consisting of consumables for each pasteurization batch, will depend greatly on the number of samples to be analyzed. In this regard, the HTST method allows us to pasteurize more liters of milk and to make mixtures of milk from several donors, reducing the number of samples to be analyzed for each batch; consequently, variable costs were reduced by almost half compared to the HoP method.

The size and volume of activity of each hospital where the bank resides is also decisive in this respect. The greater the volume of purchases of consumables, the lower the price and therefore the cheaper the variable cost associated with obtaining each liter of pasteurized milk, regardless of the pasteurization method.

Another aspect to highlight in terms of variable cost has to do with the cost of the microbiology service. In some hospitals this cost is assumed as part of the general costs, in others a cost per sample may be charged. For large volumes of samples to be processed, a timetable for the dedication of professionals and minimum fixed costs should be negotiated between the management of the milk bank and the microbiology service, so that the activity can be planned, and the processing of pasteurized milk cannot suffer delays in its supply to patients.

The total cost associated with health care professionals was the same (€ 94,880.00) for both pasteurization methods in a newly opened HMB since it would be necessary to hire at least two laboratory technicians and a medical supervisor. However, the opportunity cost of these health care professionals when the HMB was active is lower when HTST pasteurization was chosen versus HoP (€ 84.00 versus € 191.00) since the time required from these health care professionals was different throughout the subprocesses. Thus, HoP requires a great deal of time from both laboratory technicians for the preparation of the aliquots and the four complete pasteurization cycles (heating and cooling) in the thermal baths. On the other hand, HTST pasteurization, being a continuous treatment, allows the supervisor and a technician to perform other tasks. In addition, the HTST method is automated, reducing human handling and avoiding microbiological contamination of the milk, which in turn avoids the costs associated with milk losses. However, as it is a continuous process and involves the loss of a very precious biological fluid, the optimization of the HTST pasteurization process to significantly reduce said losses is a priority that is being carried out. This improvement will allow a greater volume of pasteurized DHM to be obtained at the same cost as described.

Another advantage of HTST pasteurization is that it allows a greater volume of milk to be pasteurized daily. In this study, a maximum amount of 10 L per day was established using HoP. In contrast, an HTST system could triple or quadruple this amount, although a hypothetical scenario of a maximum of 20 L was considered (scenario 3). Under these conditions, HTST pasteurization represents a minimization of costs compared to HoP for the analyzed years. In year 1, the unit cost of pasteurized milk using HTST pasteurization was € 74.93.00 versus € 85.86.00 using HoP, which represents a 13% cost minimization. In year 2, this cost was further reduced by 29.6%. This difference is likely to be maintained in the future once the first investments have been amortized.

In this regard, the creation of regional HMBs associated with satellite centers and located in public hospitals, such as the one analyzed in this study, is more cost-effective than small HMBs in each hospital. Affumicato et al. reported that a satellite center depending on the Milk Bank of Virgen de las Nieves Hospital in Granada, Spain implies savings of € 88,852.00 in equipment and € 24,572.00 per year in maintenance compared to an independent milk bank [26]. Therefore, if a newly opened HMB intends to be regional to pasteurize large volumes of DHM and meet the needs of the largest number of preterm or sick newborns, the implementation of HTST pasteurization is also a more cost-effective option in the long term than the traditional HoP method.

The main limitation of our economic analysis is the lack of results comparing the impact of both pasteurization methods on clinically significant endpoints (e.g., infection incidence, NEC, etc.). Currently, our research group is conducting a clinical trial to study the frequency of nosocomial infection in infants weighing less than 1000 g at birth when milk is pasteurized by HTST vs. the Holder method (ClinicalTrials.gov Identifier: NCT04424667). The results of this study could allow the development of a cost-effectiveness analysis that facilitates the decision of newly opened or active HMBs to implement one pasteurization method or another based on health benefits, if any.

In addition, more research and development of analytical knowledge translation tools may help educate policy-makers on the economic benefits to children, women and caregivers, households, governments and societies as a whole related to breastfeeding and nutrition. The Cost of Not Breastfeeding Tool is an example of how the big data revolution in global health can support stronger advocacy and meaningful policy change [27].

## Conclusions

The data obtained in this study may be useful for newly opened or active HMBs to select HTST pasteurization versus the traditional HoP method. This alternative heat treatment ensures the microbiological safety of DHM, reduces handling during processing, and provides greater retention of biologically and nutritionally active compounds present in the milk. Although this technology requires a high initial investment in equipment, it provides a significant minimization of production costs in the long term, pasteurizes large quantities of donor milk per working day and achieves a more efficient management of the time of the health care professionals in charge of the bank’s operation.

## Data Availability

The datasets used and / or analyzed during the current study are available from the corresponding author on reasonable request.

## Abbreviations

DHM: Donor human milk
HMBs: Human Milk Banks
HoP: Holder pasteurization
HTST: High-Temperature Short-Time
MOM: mother’s own milk
NEC: necrotizing enterocolitis
PVL: selling price in Spain
